# Development and evaluation of high‐density Axiom^®^ *CicerSNP* Array for high‐resolution genetic mapping and breeding applications in chickpea

**DOI:** 10.1111/pbi.12836

**Published:** 2017-10-31

**Authors:** Manish Roorkiwal, Ankit Jain, Sandip M. Kale, Dadakhalandar Doddamani, Annapurna Chitikineni, Mahendar Thudi, Rajeev K. Varshney

**Affiliations:** ^1^ International Crops Research Institute for the Semi‐Arid Tropics (ICRISAT) Hyderabad India; ^2^ School of Agriculture and Environment & Institute of Agriculture The University of Western Australia Crawley Perth Australia

**Keywords:** chickpea, drought, genotyping, molecular breeding, QTLs, SNP array

## Abstract

To accelerate genomics research and molecular breeding applications in chickpea, a high‐throughput SNP genotyping platform ‘Axiom^®^ *CicerSNP* Array’ has been designed, developed and validated. Screening of whole‐genome resequencing data from 429 chickpea lines identified 4.9 million SNPs, from which a subset of 70 463 high‐quality nonredundant SNPs was selected using different stringent filter criteria. This was further narrowed down to 61 174 SNPs based on *p*‐convert score ≥0.3, of which 50 590 SNPs could be tiled on array. Among these tiled SNPs, a total of 11 245 SNPs (22.23%) were from the coding regions of 3673 different genes. The developed Axiom^®^ *CicerSNP* Array was used for genotyping two recombinant inbred line populations, namely ICCRIL03 (ICC 4958 × ICC 1882) and ICCRIL04 (ICC 283 × ICC 8261). Genotyping data reflected high success and polymorphic rate, with 15 140 (29.93%; ICCRIL03) and 20 018 (39.57%; ICCRIL04) polymorphic SNPs. High‐density genetic maps comprising 13 679 SNPs spanning 1033.67 cM and 7769 SNPs spanning 1076.35 cM were developed for ICCRIL03 and ICCRIL04 populations, respectively. QTL analysis using multilocation, multiseason phenotyping data on these RILs identified 70 (ICCRIL03) and 120 (ICCRIL04) main‐effect QTLs on genetic map. Higher precision and potential of this array is expected to advance chickpea genetics and breeding applications.

## Introduction

Ongoing climatic changes that are affecting crop yields drastically and increasing food demand from ever increasing world population would certainly make the scenario worst by 2050 when the world population would be crossing the count of 9 billion. In order to feed this huge population and meet the future demands, it has become necessary to understand the factors involved in affecting the growth and yield of the crops. Chickpea (*Cicer arietinum* L., 2*n* = 16) is the second most important grain legume after common bean and third important pulse grown on low‐input marginal lands and known to play a key role in food and nutritional security globally (Jukanti *et al*., 2012). Besides its ability to fix atmospheric nitrogen that improves the soil nutritional profile, chickpea further plays a pivotal role in yield and nutritional profile of other crops when involved in crop rotation programme (Gan *et al*., 2010). Currently, global chickpea acreage in 56 countries covers more than 13.98 mha area and accounts for a production of 13.23 million tons annually (FAO 2014). With the impact of various abiotic and biotic stresses, current average chickpea productivity is <1 t/ha, which is far less than its actual potential yield of 6 t/ha under optimum growing conditions. Among these abiotic stresses, terminal drought alone is known to reduce the annual production by 40% (Ahmad *et al*., 2005). During the last five decades, conventional breeding efforts could enhance the chickpea productivity from 0.6 t/ha (1960) to 0.9 t/ha (2014) (FAO 2014). However, this increase is not enough to meet the significantly increasing global demand. Therefore, intervention of modern technology for enhancing the crop productivity has become essential. Success stories of application of genomic approaches in enhancing the production among different crop species confirm the potential of genomics‐assisted breeding (GAB) (Kole *et al*., 2015; Varshney *et al*., 2005). Inspired by the success of GAB, chickpea community also started to deploy markers for developing superior lines and were able to develop improved lines with enhanced yield under rainfed conditions in JG 11 background (Varshney *et al*., 2013a) and lines resistant to fusarium wilt and ascochyta blight in the genetic background of C 214 (Varshney *et al*., 2014a). Very recently, efforts to deploy genomic selection for yield‐related traits in chickpea have also been initiated (Roorkiwal *et al*., 2016).

Advances in next‐generation sequencing (NGS) and high‐throughput genotyping technologies have enabled the use of these technologies at lower cost and offered opportunities to deliver high‐throughput data to capture millions of variations in genome level. With the availability of draft genome sequence (Varshney *et al*., 2013b) and large‐scale resequencing efforts (Thudi *et al*., 2016a,b), large number of single nucleotide polymorphism (SNP) markers have become available. SNPs, due to their abundance at genome‐wide level, biallelic and reproducible nature, are considered to be the most desirable, precise and efficient tools for developing high‐density genome scans (Gupta *et al*., 2008; Wang *et al*., 1998). SNPs are often efficiently used to determine the functional relevance of genomic regions/candidate genes responsible for complex traits in many crop plants such as rice (Parida *et al*., 2012), maize (Pace *et al*., 2015; Riedelsheimer *et al*., 2012; Weber *et al*., 2008) and barley (Mora *et al*., 2016).

In order to use huge genomic resources for breeding applications, there is a dire need of low‐cost, high‐throughput genotyping platforms to construct high‐density genetic maps and undertake QTL analysis. Recent development in the array technology has brought down the cost of high‐throughput genotyping platforms, thus making it accessible to most of the researchers and breeding communities engaged in genetic studies and crop improvement applications. SNP genotyping platforms can be used for genetic diversity studies, foreground selection, fine mapping, association mapping, genomic selection and evolutionary studies. As a result, SNP arrays with large number of SNPs distributed throughout the genome have been developed and used for various applications including association mapping, genetic diversity and genomic selection in several agronomically important crops (see Rasheed *et al*., 2017).

With the availability of high‐quality chickpea genome (Varshney *et al*., 2013b) and various large‐scale resequencing projects (Thudi *et al*., 2016a,b; unpublished data), millions of SNPs have become available in chickpea. This information can be further exploited to access the genome‐wide sequence variations, which can help to expedite the process of development of improved lines in chickpea. With an objective to utilize SNP resource for chickpea improvement, the current study deals with the development of a high‐density SNP array with genome‐wide distributed SNPs. This array has been used for genotyping two chickpea intraspecific recombinant inbred line (RIL) populations to assess the utility of the developed SNP array in genetics and breeding applications. In brief, Axiom^®^ *CicerSNP* Array is expected to provide a solid foundation for establishing the high‐throughput genotyping, which is of great importance for research as well as breeding applications.

## Results

### SNP selection and Axiom^®^ *CicerSNP* Array design

The resequencing data generated from 429 genotypes were aligned to the chickpea reference genomes for SNP discovery. The alignment of sequencing data resulted in the identification of total 4.9 million SNPs distributed across genome.

**Figure 1:**
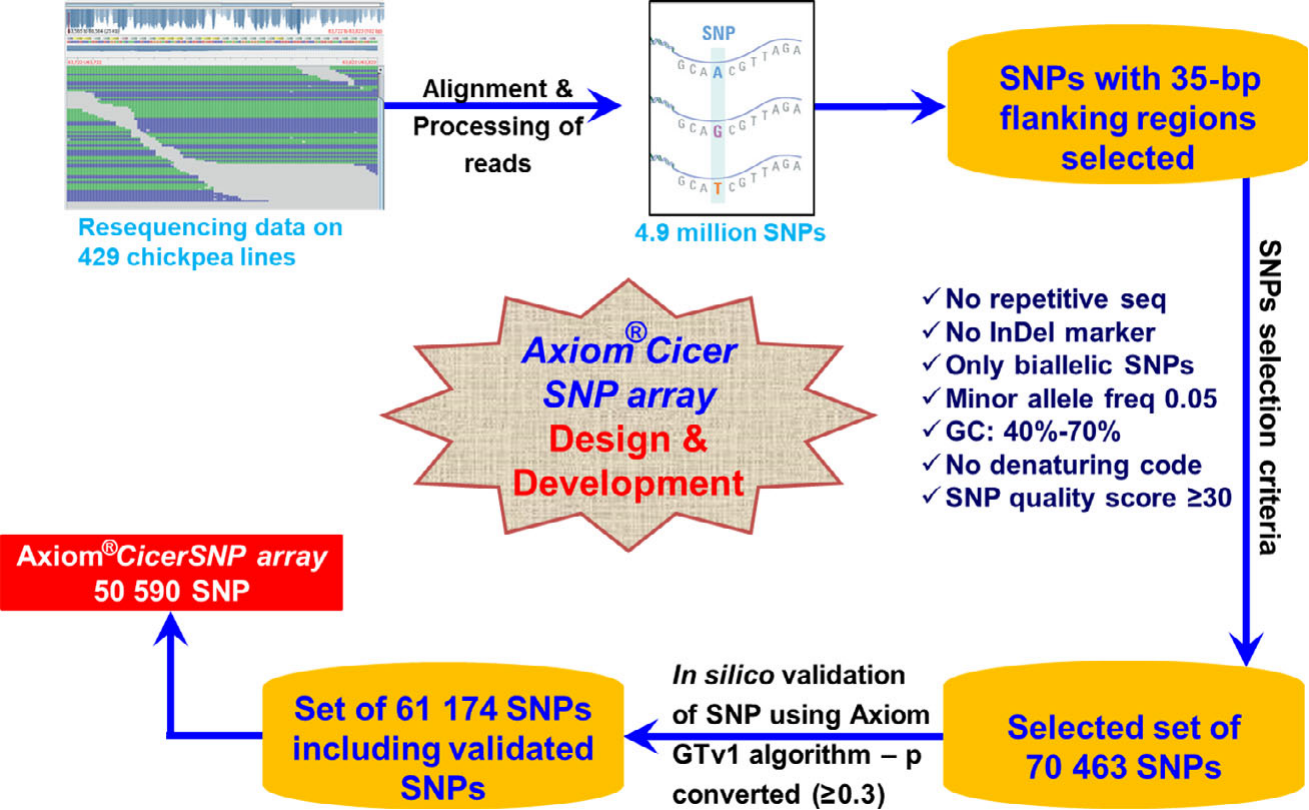
Design and development workflow of Axiom^®^ *CicerSNP* Array. Tiling to array included three major steps (i) SNP detection, (ii) SNP selection that included quality filtration and SNP validation and (iii) final tiling on array.

As described in the ‘Methods’ section and Figure**** 1, after applying seven filter criteria on identified SNPs from resequencing data, a total of 70 463 high‐quality SNPs were selected. Further on the basis of p‐convert value of ≥0.3**,** generated by *in silico* validation of SNPs using Axiom GTv1 algorithm, 61 174 SNPs were selected for further processing. Finally, a custom array consisting of a total of 50 590 SNPs was developed that includes final data set of high‐quality SNPs along with previously validated 70 and 32 SNPs from QTL‐seq (Singh *et al*., 2016) and skim sequencing (Kale *et al*., 2015), respectively. In the selected set of SNPs, 22.23% SNPs were from coding region of the genome and spanning across 3673 different genes of eight chickpea linkage groups.

### Genome‐wide distribution of selected SNPs of Axiom^®^ *CicerSNP Array*

**Figure 2:**
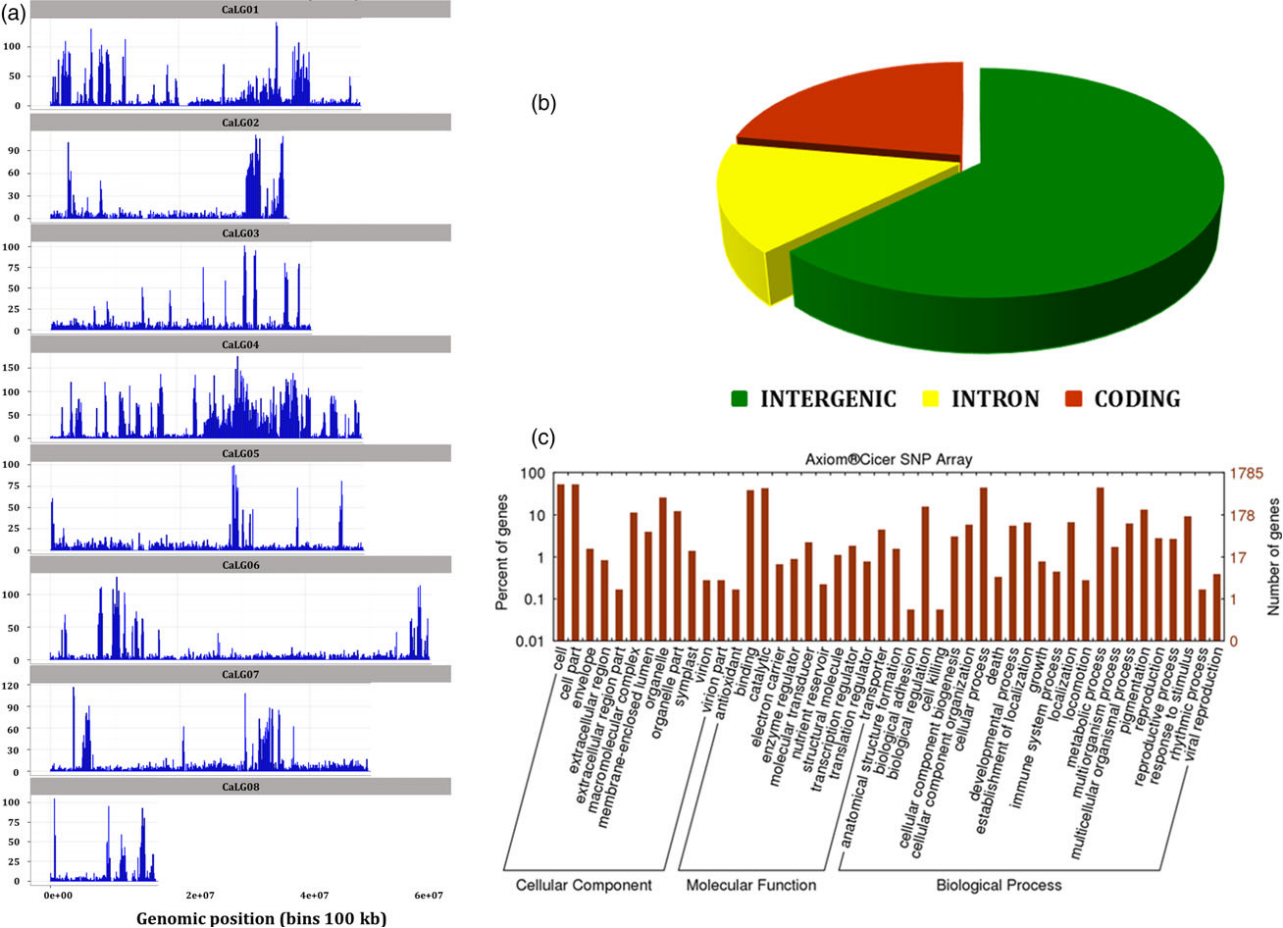
Summary of distribution of SNPs selected for *Axiom* ^®^ *CicerSNP* Array: (a) SNPs distribution along the eight linkage groups of chickpea; (b) distribution of SNPs in different genomic regions of chickpea genome.

Selected 50 590 SNPs cover all the eight pseudomolecules of chickpea and provide a good representation of whole chickpea genome (Figure**** 2a,b), with an average of 6323 SNPs/linkage group (LG) (average distance of 6.86 Kb). Maximum number of SNPs have come from CaLG04 (16 772; 33.15%), while minimum number of SNPs have come from CaLG08 (1888; 3.73%). As the upstream and downstream regions are not annotated in chickpea genome assembly, these were not considered while predicting SNP effect using SnpEff software. With respect to genomic region in the Axiom^®^ *CicerSNP* Array, as expected maximum number of SNPs have come from intergenic regions (31 653; 62.57%) followed by 11 245 SNPs (22.23%) from coding regions and 7688 SNPs (15.20%) from intronic regions (Figure 2b)**.** The SNPs from coding region cover synonymous coding (8902; 17.60%), nonsynonymous coding (2267; 4.48%), stop gained (23), synonymous stop (14), stop lost (10), synonymous start (8), splice site donor (8), splice site acceptor (7) and start lost (6).

Functional annotations of SNPs resulted in detailed classification of involvement of SNP‐carrying genes in (i) biological process, (ii) molecular function and (iii) cellular component (Figure**** 2c). Under ‘molecular function’ category, the majority of SNP‐carrying genes were involved in binding and catalytic activities. Under ‘biological process’ category, large number of SNP‐carrying genes were found to be involved in ‘cellular and metabolic processes’ and for ‘cellular component’ category, majority of the genes annotated were found to be related to classes ‘cell’ and ‘cell parts’.

### Validation and deployment of the Axiom^®^ *CicerSNP* Array

**Figure 3:**
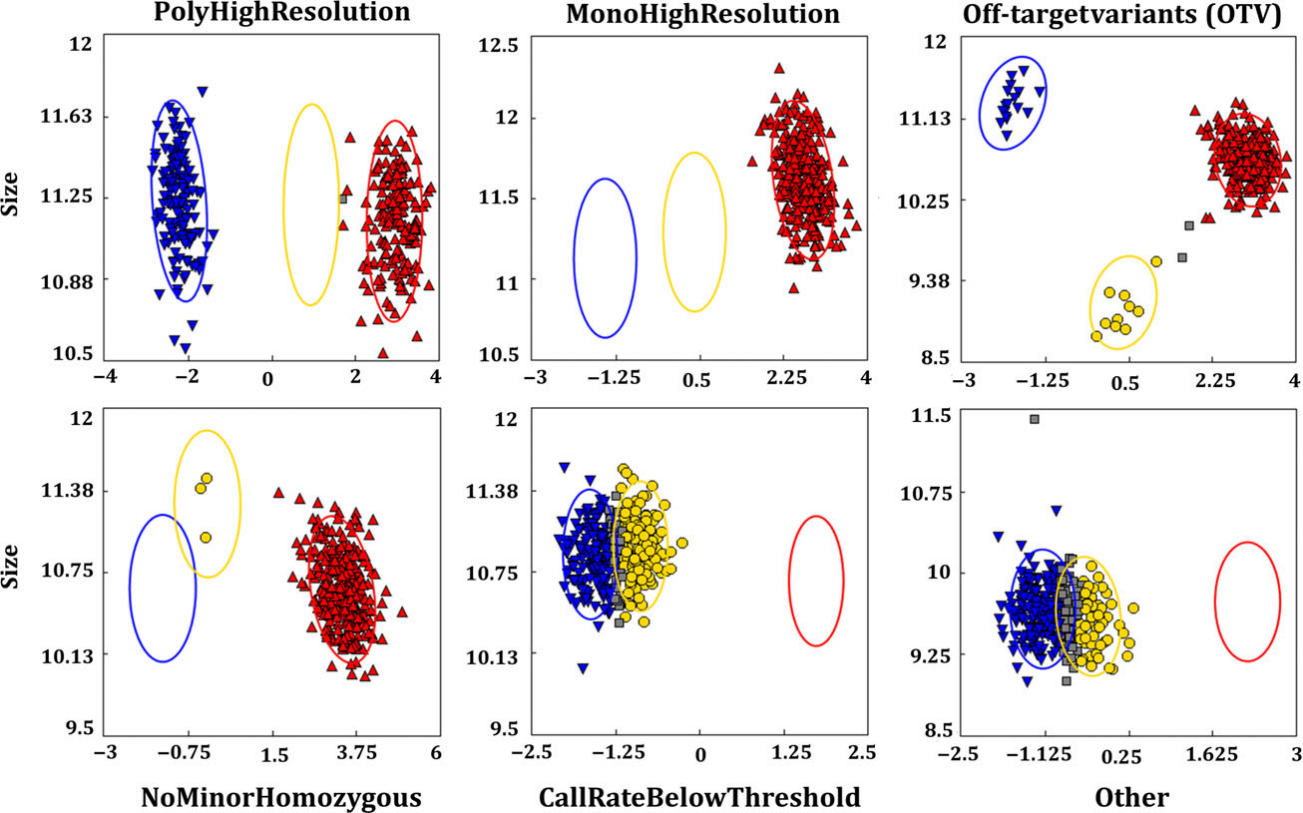
SNP classification. Different categories of SNPs identified as a result of allele calling from Axiom Analysis Suite (1.1.0.616).

Two different RIL populations, namely ICCRIL03 and ICCRIL04, were used to demonstrate the application of Axiom^®^ *CicerSNP* Array. ICCRIL03 consisting of 245 lines was genotyped with the developed Axiom^®^ *CicerSNP* Array. Three samples were removed from the analysis due to below‐threshold standard dish quality control check (DQC < 0.82). Seven additional samples were removed due to a lower call rate (<97%). An initial performance validation of the Axiom^®^ *CicerSNP* Array followed the Axiom best practices genotyping workflow. SNPs were classified as described in ‘Methods’ section (Figure**** 3), and results are summarized in Table 1. Overall, 25.89% SNPs were found to fall in ‘PolyHighResolution’ category, whereas 45.7% SNPs were found to fall in ‘MonoHighResolution’ category. A summary of the distribution of all SNPs in the different classes and on different LGs is shown in Table S1.

**Table 1:** Summary of SNP data generated in two RIL populations using *Axiom* ^*®*^ *CicerSNP Array*

Categories	ICCRIL03		ICCRIL04	
	No. of SNP markers	%	No. of SNP markers	%
MonoHighResolution	23 120	45.70	18 998	37.55
PolyHighResolution	13 099	25.89	18 059	35.70
Other	7821	15.46	7548	14.92
NoMinorHom	2713	5.36	2950	5.83
HomHomResolution	1716	3.39	1632	3.23
Off‐target variants (OTV)	990	1.96	347	0.69
CallRateBelowThreshold	806	1.59	729	1.44
AAvarianceY	139	0.27	88	0.17
BBvarianceX	102	0.20	135	0.27
BBvarianceY	71	0.14	79	0.16
AAvarianceX	13	0.03	25	0.05

In the case of ICCRIL04, consisting of 230 lines, one sample was excluded from the analysis due to lower dish quality control check and 15 samples were removed due to a lower call rates. Overall, 35.7% SNPs were found to fall in ‘PolyHighResolution’ category, whereas 37.55% SNPs were found to fall in ‘MonoHighResolution’ category (Table 1 and Table S2).

### Construction of genetic maps

**Figure 4:**
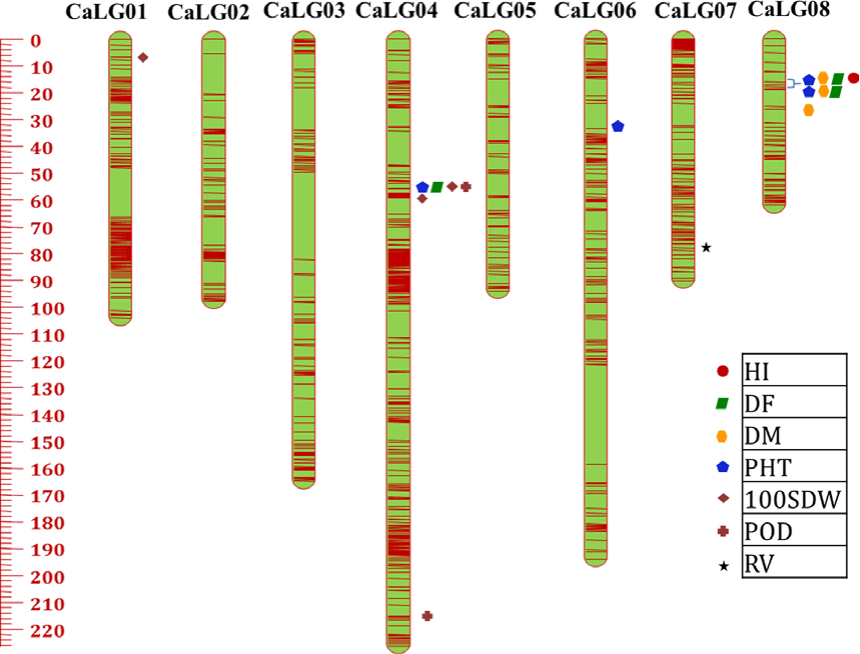
High‐resolution genetic map comprising eight linkage groups (CaLG01–CaLG08), with marked major QTL clusters, using 13 679 SNP markers for ICCRIL03 (ICC 4958 × ICC 1882).

Genotypic data generated using Axiom^®^ *CicerSNP* Array were used for the construction of genetic maps for ICCRIL03 and ICCRIL04 populations. For ICCRIL03, a total of 15 140 high‐quality SNPs were identified, of which 14 034 showed expected 1:1 segregation at *P* ≤ 0.01. Finally, 13 679 SNPs were successfully mapped to eight linkage groups (CaLG01–CaLG08) covering 1033.67 cM (Table 2). The highest number of markers were mapped on CaLG04 (5179), while the lowest number of markers were mapped on CaLG05 (212). The distribution of marker mapped on eight linkage groups is shown in Figure**** 4. The total map distance for linkage groups varied from 62.24 cM (CaLG08) to 226.43 cM (CaLG04). The highest average marker density was observed for CaLG01, which had 25 markers/cM followed by 23 markers/cM on CaLG04. The lowest average marker density was observed for CaLG05, which had two markers per cM. In summary, the genetic map has 13 markers/cM on an average (Table 2).

**Table 2:** Features of genetic map developed for ICCRIL03 (ICC 4958 × ICC 1882) population using Axiom^®^ *CicerSNP* Array and its comparison with earlier studies

Linkage group	Axiom^®^ *CicerSNP Array* (Current study 2017)		GBS (Jaganathan *et al*., 2015)		SSRs (Varshney *et al*., 2014a, 2014b)	
	Marker loci mapped	Map Distance (cM)	Marker loci mapped	Map Distance (cM)	Marker loci mapped	Map Distance (cM)
CaLG01	2610	104.02	109	101.27	31	99.27
CaLG02	1088	97.67	90	92.16	18	78.50
CaLG03	576	164.90	90	72.78	41	28.13
CaLG04	5179	226.43	386	112.10	45	111.90
CaLG05	212	94.15	39	59.41	22	33.24
CaLG06	1709	193.99	160	104.36	36	123.08
CaLG07	1837	90.27	60	96.59	27	96.11
CaLG08	468	62.24	73	88.62	21	51.28
Total	13 679	1033.67	1,007	727.29	241	621.51
Average	1709.88	129.21	125.88	90.91	30.13	77.69

**Figure 5:**
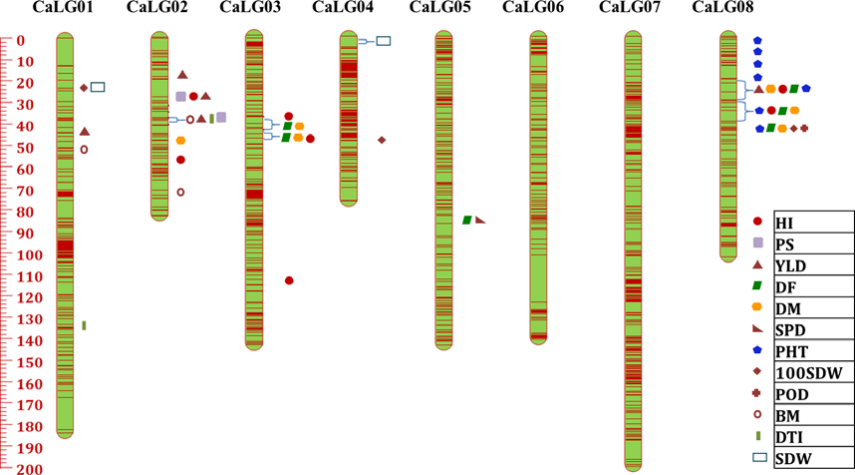
High‐resolution genetic map ICCRIL04 (ICC 283 ×  ICC 8261) with major QTL clusters constructed using 7769 SNPs.

Similarly for ICCRIL04 population, 20 018 high‐quality SNPs were identified, of which 8224 SNPs showed 1:1 segregation and 7769 SNPs could be mapped, covering 1076.35 cM across eight linkage groups (Figure 5). Maximum number of markers were mapped to CaLG01 (2001) followed by CaLG07 (1727), whereas minimum number of markers were mapped to CaLG02 (154). Highest average marker density was observed for CaLG04 (19.74 markers/cM), and minimum marker density of 1.85 markers/cM was observed for CaLG02. Overall, the map has an average of 7.22 markers/cM (Table 3).

**Table 3:** Features of genetic map developed for ICCRIL04 (ICC 283 ×  ICC 8261) population using Axiom^®^ *CicerSNP* Array and its comparison with earlier studies

Linkage group	Axiom^®^ *CicerSNP Array* (Current study 2017)		SSRs (Varshney *et al*., 2014a, 2014b)	
	Marker loci mapped	Map distance (cM)	Marker loci mapped	Map distance (cM)
CaLG01	2001	183.98	16	60.78
CaLG02	154	83.16	16	66.61
CaLG03	1063	144.19	22	69.44
CaLG04	1516	76.79	18	43.95
CaLG05	440	143.76	23	51.51
CaLG06	557	141.43	31	65.29
CaLG07	1727	200.30	24	104.92
CaLG08	311	102.74	18	70.57
Total	7769	1,076.35	168	533.07
Average	971.13	134.54	21	66.63

### QTLs for drought tolerance‐related traits

QTL analysis using genotyping and phenotyping data resulted in the identification of a large number of QTLs for five groups of drought component traits such as root traits, morphological traits, phenological traits, yield and yield‐related traits and transpiration efficiency (TE) using QTL IciMapping software. In the case of ICCRIL03, a total of 70 QTLs were identified that included 26 QTLs for phenological traits, 11 QTLs for morphological traits, 21 QTLs for yield‐related traits, 6 QTLs for drought indices, 5 QTLs for root traits and 1 QTL for carbon isotope discrimination (δ^13^C) (Table S3). However, in the case of ICCRIL04, 120 QTLs were identified, namely 48 QTLs for yield‐related traits, 41 QTLs for morphological traits, 21 QTLs for phenological traits, 8 QTLs for root traits and 2 QTLs for drought indices (Table S4). Of the 70 QTLs identified in ICCRIL03, 16 QTLs (22.86% of total QTLs identified) were located on CaLG08 followed by CaLG04 and CaLG05 (20%, 14 QTLs each). However for ICCRIL04, 33 QTLs (27.5% of total QTLs) were located on CaLG08 followed by CaLG03 with 25 QTLs (20.83%). In total, 52 major QTLs were identified across all linkage groups for morphological traits, which is quite high in comparison with previous studies (Varshney *et al*., 2014b). Of these 52 QTLs, 16 QTLs were designated as robust QTLs based on phenotypic variation explained (PVE) > 10%. QTLs for plant height (PHT, cm) were detected in both RIL populations. However, QTLs for primary branches (PBS) and secondary branches (SBS) were specific to ICCRIL03 population, and QTLs for shoot dry weight (SDW, g), plant width (PWD, cm) and plant stand (PS) were specific to ICCRIL04 population.

In the case of ICCRIL03, three QTL clusters were observed. Cluster one located on CaLG04 represented QTLs for 100‐seed weight (100‐SDW, g), days to 50% flowering (DF), PHT, pods per plant (POD), while other two clusters were located on CaLG08 responsible for DF, days to maturity (DM), harvest index (HI, %), PHT and DF, DM, PHT traits, respectively. Similarly in the ICCRIL04 population, a total of nine QTL clusters were identified on CaLG01 (one cluster: 100‐SDW, SDW), CaLG02 (two clusters: a) HI, PS, yield (YLD, g); (b) biomass (BM, g), drought tolerance index (DTI), PS, YLD), CaLG03 (one cluster for DF and DM and another cluster for DF, DM, HI), CaLG05 (one cluster: DF and seeds per pod (SPD)) and CaLG08 (three clusters: a) DF, DM, HI, PHT, YLD; b) DF, DM, HI, PHT; c) 100‐SDW, DF, DM, PHT, POD).

On the basis of location/year, QTLs were further categorized in two categories: (i) QTL for a trait appearing in more than one location was considered as ‘stable’ QTL and (ii) QTL appearing for >1 year or season was considered ‘consistent’ QTL (Tables 4 and 5). PVE for QTLs ranged from 4.8% to 66.49% in case of ICCRIL03 population and 5.49% to 39.32% in case of ICCRIL04 population (Tables S3 and S4).

**Table 4:** Detailed comparison of robust QTLs for drought tolerance‐related traits identified using high‐density genetic map in the current study with the earlier studies for ICCRIL03 (ICC 4958 × ICC 1882)

Traits	Axiom^®^ *CicerSNP Array* (Current study 2017)				GBS (Jaganathan *et al*., 2015)				SSRs (Varshney *et al*., 2014a, 2014b)			
	Total QTLs	Consistent QTLs	Stable QTLs	PVE (%)	Total QTLs	Consistent QTLs	Stable QTLs	PVE (%)	Total QTLs	Consistent QTLs	Stable QTLs	PVE (%)
Root traits												
Root length density (RLD, cm/cm^3^)	–	–	–	–	1	1	–	10.65–12.09	1	–	–	10.90
Root surface area (RSA, cm^2^)	–	–	–	–	1	–	–	11.04	1	–	–	10.26
Root volume (RV, cm^3^)	1	–	–	66.49	–	–	–	–	–	–	–	–
R–T ratio (RTR, %)	–	–	–	–	1	1	–	10.85–13.56	1	–	–	16.67
Morphological traits												
Plant height (PHT, cm)	4	–	–	10.6–13.51	9	5	3	10.05–34.57	4	2	1	10.00–30.20
Shoot dry weight (SDW, g)	–	–	–	–	3	1	–	10.78–26.91	1	1	–	13.89–17.59
Phenological traits												
Days to 50% flowering (DF)	3	2	2	14.79–34.82	3	1	1	10.86–67.71	2	1	1	10.51–26.87
Days to maturity (DM)	3	2	2	10.57–26.10	2	1	1	10.11–47.43	3	1	1	12.13–19.71
Yield–related traits												
100–seed weight (100–SDW, g)	3	2	2	11.45– 33.60	2	2	2	10.12–60.41	2	1	1	10.31–58.20
Biomass (BM, g)	–	–	–	–	3	–	–	10.11–16.63	2	–	–	10.95–21.32
Harvest index (HI, %)	1	–	–	16.05	3	1	–	10.14–25.94	3	–	–	10.67–14.36
Pods/plant (POD)	2	–	–	12.56–24.07	2	1	–	10.73–32.34	1	1	–	10.19–23.18
Seeds/pod (SPD)	–	–	–	–	3	–	–	11.09–45.40	1	–	–	42.07
Yield (YLD, g)	–	–	–	–	3	–	–	11.67–18.64	2	–	–	13.98–15.71
Drought indices traits												
Drought tolerance indices (DTI)	–	–	–	–	3	–	–	10.10–10.76	1	–	–	11.23
Total	17				39				25			

PVE, phenotypic variation explained; –, No robust QTL identified.

**Table 5:** Detailed comparison of robust QTLs for drought tolerance‐related traits identified using high‐density genetic map in the current study with the earlier studies for ICCRIL04 (ICC 283 × ICC 8261)

Traits	Axiom^®^ *CicerSNP Array* (Current study 2017)				SSRs (Varshney *et al*., 2014a, 2014b)			
	Total QTLs	Consistent QTLs	Stable QTLs	PVE (%)	Total QTLs	Consistent QTLs	Stable QTLs	PVE (%)
Morphological traits								
Shoot dry weight (SDW, g)	2	0	0	10.08–17.12	–	–	–	–
Plant height (PHT, cm)	8	2	2	11.05–17.65	2	1	–	11.27–31.32
Plant width PWD (cm)	–	–	–	–	1	–	–	15.84
Plant stand (PS)	2	–	–	12.5–16.9	–	–	–	–
Phenological traits								
Days to 50% flowering (DF)	6	3	3	10.68–39.32	4	2	1	10.66–18.97
Days to maturity (DM)	6	1	3	11.91–37.5	4	–	1	10.47–16.79
Yield‐related traits								
100‐seed weight (100‐SDW, g)	3	–	–	13.49–16.84	1	1	–	17.14–26.67
Biomass (BM, g)	3	–	–	10.24–15.52	–	–	–	–
Harvest index (HI, %)	7	2	–	10.25–35.88	2	–	–	12.06–14.04
Pods/plant (POD)	1	–	–	14.17	1	1	–	12.13–14.37
Seeds/pod (SPD)	1	–	–	15.58	–	–	–	–
Yield (YLD, g)	5	–	–	10.58–15.47	3	–	–	10.06–18.55
Drought indices traits								
Drought tolerance index (DTI)	2	–	–	18.63–28.49	2	–	–	11.27–12.12
Total	46				20			

PVE, phenotypic variation explained; –, No robust QTL identified.

### Root traits

A total of six root traits [root length density (RLD, cm/cm^3^), root dry weight (RDW, g), rooting depth (RDp, cm), root surface area (RSA, cm^2^), root volume (RV, cm^3^) and root dry weight/total plant dry weight ratio (RTR, %)] were analysed and QTLs for five traits one each for RLD, RSA, RV, RTR and RDp in ICCRIL03 with PVE ranging from 6.55% (RSA) to 66.49% (RV) were identified (Table S3). However, in case of ICCRIL04, one QTL was identified each for RLD, RSA and RDp, and 2 QTLs for RDW and 3 QTLs for RTR were identified with PVE ranging from 5.79% (RLD) to 8.08% (RDW) (Table S4). One robust QTL was observed for RV in ICCRIL03 population (Table 4), whereas no robust QTL was observed in case of ICCRIL04 (Table 5). In both the RIL populations, no consistent and no stable robust QTL was observed for root traits (Tables 4 and 5).

### Morphological traits

A total of 11 QTLs were identified in ICCRIL03 population for morphological traits that included PHT (7 QTLs), PBS (3 QTLs) and SBS (1 QTL). Overall, PVE ranged from 6.12% (PHT) to 13.51% (PHT) (Table S3). In all, four robust QTLs were observed for PHT (Table 4).

In the ICCRIL04 population, 41 QTLs for morphological traits were identified, of which 17 QTLs were for PHT, 11 QTLs for SDW, 9 QTLs for PS and 4 QTLs for PWD. PVE ranged from 5.49% (SDW) to 17.65% (PHT) among QTLs for morphological traits (Table S4). In total, 12 robust QTLs were observed including two for SDW which were found consistent as well as stable. In the case of PHT, eight robust QTLs were observed, of which two QTLs were consistent as well as stable. However, for PS, two robust QTLs were observed and none of them was stable or consistent (Table 5).

### Phenology‐related traits

For phenological traits in ICCRIL03 population, a total of 26 QTLs were identified for two traits, *viz*. DF (13 QTLs) and DM (13 QTLs), with PVE ranging from 4.8% (DF) to 34.82% (DF) (Table S3). Of total of 26 QTLs, six robust QTLs were found, three each for DF and DM. Two QTLs each from DF and DM were both consistent and stable (Table 4).

In ICCRIL04 population, a total of 21 QTLs were observed, of which 10 for DF and 11 for DM with PVE ranging from 5.59% (DF) to 39.32% (DF). A total of 12 robust QTLs were identified, six for each DF and DM (Table S4). Three QTLs were found both consistent and stable for DF. However for DM, two stable robust QTLs and one additional stable and consistent QTL were observed (Table 5).

### Yield and yield‐related traits

In total, 21 QTLs were identified for six traits, *viz*. 3 QTLs for 100‐SDW, 3 QTLs for POD, 1 QTL for SPD, 2 QTLs for BM, 8 QTLs for HI and 4 QTLs for YLD in ICCRIL03 population (Table S4). PVE of identified QTLs was ranged from 5.59% (HI) to 33.6% (SDW). Overall, six QTLs were found to be robust, two for POD, one for HI and three for 100‐SDW (of which two were both stable and consistent; Table 4).

In ICCRIL04 population, a total of 48 QTLs were identified for six traits with PVE ranging from 5.49% (YLD) to 35.88% (HI) (Table S4). There were 2 QTLs for SPD, 3 QTLs POD, 11 QTLs for HI, 6 QTLs for 100‐SDW, 13 QTLs each for BM and YLD. A total of 20 QTLs were found to be robust, five for YLD, three each for 100‐SDW and BM, one each for SPD and POD and seven for HI. Among yield traits, only two QTLs were found consistent for HI (Table 5).

### Drought indices and Transpiration efficiency

In the case of ICCRIL03, a total of six QTLs were identified, namely four QTLs for DTI and two for drought susceptibility index (DSI) with PVE ranging from 5.58% (DSI) to 8.13% (DTI) (Table S3). However, in the case of ICCRIL04, only two QTLs for DTI were observed and both were robust (Table 4). Overall, PVE for these QTLs in ICCRIL04 population ranged from 18.63% to 28.49% (Table S4). No QTL for drought indices was found stable and/or consistent. In case of carbon isotope discrimination (δ^13^C) as a surrogacy trait for transpiration efficiency (TE), only single QTL was identified in ICCRIL03 with PVE of 5.46% (Table S3), while in the case of ICCRIL04, δ^13^C was not measured.

## Discussion

Molecular markers have been widely used for genetic diversity assessment, evolutionary and mapping studies (Varshney *et al*., 2007). Applicability of markers in breeding mainly relies on cost, ease of automation and precision, making SNPs an indispensable choice. Rapid progress in NGS technologies during last decade enabled massive sequence data output at low cost in very less time period (Thudi *et al*., 2012).

Due to limited availability of genomic resources until past decade, chickpea was considered to be an ‘Orphan Crop’ (Varshney *et al*., 2012). Chickpea improvement efforts using conventional breeding were able to enhance the chickpea productivity but were not enough to meet increase in the global demand. Availability of large‐scale genomic resources and markers has offered an opportunity to utilize GAB for crop improvement to enhance the rate of genetic gain (Varshney *et al*., 2005). The past decade has witnessed tremendous application of GAB to critically address some of the major issues in legume crops (Varshney *et al*., 2013c).

For successful and effective deployment of GAB to develop superior chickpea varieties, it becomes important to tap the variations existing in the genome by successful mapping and tagging these variations for agronomically important traits. Success of GAB depends on the level of marker precision and cost for genotyping. In the case of chickpea, different SNP genotyping platforms have been developed for various applications (Gujaria *et al*., 2011; Hiremath *et al*., 2012; Roorkiwal *et al*., 2013, 2014).

In recent past, NGS‐based technologies have been effectively used for genome sequencing and resequencing, enabling the identification of millions of SNP markers in chickpea (Kale *et al*., 2015; Thudi *et al*., 2016a,b). SNP genotyping arrays are user‐friendly and cost‐effective for generation and analysis of genotyping data. Considering the utility of these arrays, the present study focused on the development of high‐density SNP array and its utility for genetics and breeding applications in chickpea. In brief, whole‐genome resequencing of 300 lines of the reference set and >100 lines of elite chickpea varieties at 5–13× coverage led to the generation of >4.9 million SNPs. With the application of different filter criteria ensuring distribution of SNPs across all the eight linkage groups and with further inclusion of SNPs from skim sequencing, a high‐quality ‘Axiom^®^ *CicerSNP* Array’ was developed. High‐density Axiom^®^ *CicerSNP* Array with 50 590 high‐quality nonredundant SNPs produced 95.92% and 93.04% sample success rate, and average QC call rate of 99.81, among two chickpea intraspecific populations ICCRIL03 and ICCRIL04, respectively. The total missing data in case of ICCRIL03 population was 0.5% and 0.4% in ICCRIL04 population. On an average, the triplicates showed >99% concordance among the results (includes all calls for triplicates and minimum duplicates).

Chickpea crop improvement efforts are severely affected by the narrow genetic diversity among cultivated chickpea genepool. Some of the previously generated intraspecific genetic maps with only few hundred markers also indicate the narrow genetic base in chickpea (Gaur *et al*., 2011; Jamalabadi *et al*., 2013; Millàn *et al*., 2010; Radhika *et al*., 2007; Varshney *et al*., 2014b). For instance, Varshney *et al*. (2014b) after screening ~3000 markers could find only couple of hundred polymorphic markers and ultimately were able to map 241 and 168 markers on ICCRIL03 and ICCRIL04, respectively. With the availability of NGS technology, Jaganathan *et al*. (2015) using GBS approach could identify 828 novel SNPs and were able to integrate additional 743 SNPs to develop a genetic map with 1007 mapped markers spanned around 727.29 cM. However, the current study by using SNP array successfully mapped 13 679 markers spanning around 1033.67 cM (Table 2). Similarly, another advanced map was developed in the current study using 46‐fold more markers (7769) spanning 1076.35 cM map distance in comparison with genetic map constructed by Varshney *et al*. (2014b) for ICCRIL04 (Table 3). Varying levels of marker densities were recorded for different LGs in both the maps and the average intermarker distances were 0.08 cM and 0.14 cM in the case of ICCRIL03 and ICCRIL04, respectively. Genetic maps generated in current study will not be only useful in ordering future genetic maps but also a higher marker density obtained here will aid in the selection of appropriate markers for various molecular breeding applications.

QTL analysis includes identification of markers/genomic regions, associated with the genetic variation influencing economically important traits (Mackay *et al*., 2009). Markers associated with QTLs aid in improving the accuracy of genetic selection by identifying favourable genotypes. Use of Axiom^®^ *CicerSNP* Array on two RIL populations resulted in identifying a large number of QTLs for five groups of drought tolerance‐related traits such as root traits, morphological traits, phenological traits, yield and yield‐related traits and TE. In total, 70 QTLs for ICCRIL03 and 120 QTLs for ICCRIL04 were identified. In the case of ICCRIL04, more than twofold robust QTLs (46) were identified in comparison with the previous study (Varshney *et al*., 2014b). In the present study, 13 main‐effect QTLs for root traits were identified. Unlike our previous study (Varshney *et al*., 2014b) where 18 main‐effect QTLs for root traits were observed distributed all over the linkage group except for CaLG02, we observed the absence of main‐effect QTLs for root traits on CaLG01, CaLG02 and CaLG06.

While analysing for both RIL populations (ICCRIL03 and ICCRIL04), common QTLs were identified for four root traits (RLD, RSA, RTR and RdP). However, it was interesting to note that except for RLD, QTLs for these traits were identified on different linkage groups in the RIL populations. Additionally, QTLs for RV and RDW were found only in ICCRIL03 and ICCRIL04, respectively. The possible reason behind this contrasting identification of QTLs in two RIL populations could be either the presence of several small‐effect QTLs or break of robust QTLs into many small‐effect QTLs due to higher number of markers in the current study.

SNP arrays have been used for the validation of genome sequence assembly by comparing genetic position with the physical position (Sim *et al*., 2012). As an application of the Axiom^®^ *CicerSNP* Array, while working on different mapping populations comparison of genetic with physical positions of the SNP can be used for further validation and improvement of assembled chickpea genome. Some studies in maize and apple have clearly shown the possibility of improvement of genome assembly using large number of markers by applying array‐based genotyping for high‐resolution genetic mapping (Bianco *et al*., 2016; Ganal *et al*., 2011). In addition, high‐density SNP arrays have also been used for various breeding applications and high‐resolution genetic mapping using genome‐wide association studies (GWAS). Several SNP arrays including medium‐ to high‐throughput have been developed and used for GWAS and breeding applications in groundnut (Pandey *et al*., 2017), rice (Chen *et al*., 2014; McCouch *et al*., 2010; Singh *et al*., 2015; Yu *et al*., 2014) and wheat (Maccaferri *et al*., 2015).

From the cost perspective, SNP array platforms are found to be efficient and cost‐effective in comparison with other genotyping platforms. In the case of chickpea, 828 unique SNPs were identified across 208 RILs using GBS with a cost US $40–45 per sample (Jaganathan *et al*., 2015). Similarly, application of skim sequencing costing about US $100, resulted in the identification of >80 K SNPs across 232 lines (Kale *et al*., 2015). However, use of the Axiom^®^ *CicerSNP* Array can provide genotyping data for 50 590 SNPs for a cost of US $52 per sample as per the negotiated agreement between Affymetrix and ICRISAT for the chickpea community. Therefore, SNP array developed in the present study has a potential to generate low‐cost data points. Furthermore, SNP arrays produce robust and reliable data with less missing values, in comparison with GBS where the presence of large proportion of missing values and the presence of false homozygotes can be observed (Bianco *et al*., 2016). It is also important to mention that SNP array data are generated in simple allele calls and can directly be used for further analysis, suggesting minimal computational skills requirement. However, advanced computational skills are required to handle GBS and skim sequencing data, to analyse the generated data that further aid to the postprocessing cost to the data analysis (Bajgain *et al*., 2016). Hence based on these parameters, it can be concluded that the Axiom^®^ *CicerSNP* Array developed under this study will be a valuable tool to dissect agronomically important traits and for various molecular breeding applications in chickpea. Implementation of developed array in ongoing genomic selection attempts in chickpea (Roorkiwal *et al*., 2016) will further enhance the precision and accuracies of the genomic predictions.

In summary, the SNP array developed in this study is a most awaited genomic resource for global chickpea research community and will eventually accelerate the process and precision of understanding trait genetics and molecular breeding for chickpea improvement.

## Methods

### Plant material and phenotypic evaluation

Two different RIL populations, namely ICCRIL03, developed by crossing ICC 4958 (a drought‐tolerant genotype) and ICC 1882 (a drought‐sensitive genotype), and ICCRIL04 generated by crossing ICC 8261 (a drought‐tolerant genotype) and ICC 283 (a drought‐sensitive genotype), were used in this study.

Genomic DNA from parental genotypes and RILs were isolated using high‐throughput mini‐DNA extraction (Cuc *et al*., 2008). Estimation of quality and quantity of DNA was made using spectrophotometer (Shimadzu UV160A, Japan).

Phenotyping data were collected for 20 different drought‐tolerant traits that included six root traits, six yield and yield‐related traits, five morphological traits, two phenological traits and one physiological trait for both the RIL populations (ICCRIL03 and ICCRIL04). These datasets have been described in earlier studies (Varshney *et al*., 2014b).

### SNP identification and array design

In order to select the SNPs for array, the resequencing data available from a diverse set of 429 chickpea accessions from other studies were used (unpublished). SNPs from earlier studies were filtered using ‘in‐house’ multiple sequence alignments scripts and SNPs with no other SNP in the flanking 35 bp on both sides were selected. These selected SNPs were then subjected to seven different kinds of filters, namely (i) no SSR in flanking region, (ii) no Indel in flanking region, (iii) only biallelic nature SNPs, (iv) minor allele frequency of 0.05, (v) GC% to be 40%–70%, (vi) no denaturing code permitted in the region and (vii) SNP quality score ≥30 to select best SNPs for the array. High‐quality SNPs resulted from these assorted criteria were further subjected to *in silico* validation that involved preliminary screening of each selected SNP on the basis of p‐convert values generated using Affymetrix power tool (APT) AxiomGTv1 algorithm to ensure a high‐quality final array. Probability of SNP conversion represented by p‐convert value was predicted using random forest model for both forward and reverse probes of each SNP. The model takes into account factors such as sequence of the probe, binding energy and expected degree of nonspecific hybridization to multiple genomic regions. High p‐convert values of probes reflect high probability to convert on the SNP array. In addition, few SNPs selected as part of earlier studies such as results of skim sequencing (Kale *et al*., 2015) and QTL‐seq (Singh *et al*., 2016) were also included in the final list of SNPs used for array imprinting.

### Genotyping with Axiom^®^CicerSNP Array

In order to utilize the developed Axiom^®^ *CicerSNP* Array, high‐quality DNA extracted from fresh leaves of both the populations, ICCRIL03 with 245 lines and ICCRIL04 with 230 lines along with parental genotypes, were used for genotyping. In brief, high‐quality total genomic DNA (100 ng) was randomly fragmented (25–125 bp) and fragmented DNA was purified before hybridizing with Axiom^®^ *CicerSNP* Array. Under stringent conditions, nonspecific random ligations bound to targets were washed off. In order to identify multicolour ligation event at the array surface which points towards polymorphic nucleotide, array was stained, imaged and processed using GeneTitan^®^ Multi‐Channel (MC) instrument to generate the data.

Allele calling was performed using generated ‘*.CEL files*’ with Axiom Analysis Suite (1.1.0.616). Axiom best practice workflow was followed in order to get high‐quality genotyping results (Figure 1). Samples with DQC < 0.82 and QC call rate <97% were not considered for further analysis. SNP QC was performed under default parameter for diploid species type, that is cr‐cutoff ≥97; fld‐cutoff ≥3.6; het‐so‐cutoff ≥−0.1; het‐so‐otv‐cutoff ≥−0.3; hom‐ro‐1‐cutoff ≥0.6; hom‐ro‐2‐cutoff ≥0.3. The whole data were analysed at an ‘Inbred penalty score’ of 12. The results generated were further processed using the SNPolisher package that classifies SNPs into different classes as per Bassil *et al*. (2015). These different classes include MonoHighResolution (SNPs with genotyping data with QC pass but monomorphic across the genotypes studied), PolyHighResolution (genotyping data passed all QC with polymorphic SNPs), NoMinorHom (data passed all QC but only two clusters could be observed), Off‐Target Variant (data with low‐intensity cluster resulted from mismatches between the probe and the sequences for that group of genotypes) and Other (SNP with genotyping data with no clear cluster pattern and could not be assigned any of the other classes). PolyHighResolution SNPs were further filtered out for reproducibility and variance. In addition, markers with complex genotypes causing cluster splits are further filtered into additional classes using ‘Variance’ filters.

In order to identify putative function of SNP‐containing genes, similarity search with a cut‐off value of ≤1E‐5 was performed against existing NR protein sequences in the public database. In order to determine the involvement in particular biological function, these SNP‐containing genes were annotated based on the Gene Ontology (GO) terms associated with the blast results using Blast2GO software (Conesa *et al*., 2005) that performs BLASTX analysis followed by mapping and annotation step.

### Genetic map construction

The genotyping data obtained were used for genetic map construction. Initially, SNPs showing contrasting alleles between the parental lines were selected. A chi‐squared test was then conducted for each SNP with a null hypothesis that two alleles at a locus segregate with a ratio of 1:1 in RIL population. SNPs showing significant deviation from 1:1 ratio (*P* < 0.01) were removed from further analysis. The genetic map was constructed from the selected SNPs using R/qtl program (Broman *et al*., 2003) as follows: first, the preliminary map was constructed using ‘estimate.map’ function and duplicate markers were removed. The genetic map was constructed using remaining markers with maximum recombination frequency cut‐off of 0.35 and minimum LOD value of 6. The marker order was confirmed using ripple function, while Kosambi function was used to calculate the map distance. The Mapchart 2.2 software (Voorrips, 2006) was used to visualize the final genetic map.

### QTL analysis

The QTL analysis was carried out using QTL ICiMapping V3.3 software (Meng *et al*., 2015). For this, the genotyping data obtained as a result of application of Axiom^®^ *CicerSNP* Array in the current study and the phenotyping data for 20 drought tolerance‐related traits (as mentioned in Varshney *et al*., 2014b) were used. Composite interval mapping was carried out using ICIM‐ADD mapping method keeping other parameters as default. The LOD threshold was set using 1000 permutations and *P* value ≤0.05. The results obtained were compared with earlier studies to check the accuracy and further refinement.

## Conflict of interest

The authors declare no conflict of interest.

## Author contributions

M.R. performed most of the experiments; D.D. and M.R. selected and filtered SNPs for designing array; S.M.K., A.J. and M.R. analysed genotyping data and conducted genetic analysis; A.C. and M.T. contributed genetic material and sequencing data; M.R., A.J. and R.K.V. interpreted the results and wrote the manuscript; R.K.V. conceived, designed and supervised the study.

## Supporting information


**Table S1** Linkage group wise SNP data summary in ICCRIL03 (ICC 4958 × ICC 1882) population.

**Table S2** Linkage group wise SNP data summary in ICCRIL04 (ICC 283 × ICC 8261) population.

**Table S3** Details on QTLs identified for the 17 traits and two drought indices in ICC 4958 × ICC 1882 population.

**Table S4** Details on QTLs identified for the 17 traits and one drought indices in the ICC 283 × ICC 8261 population.
